# The impact of perceived social support on psychological capital of firefighters: the chain mediating role of exercise self-efficacy and psychological resilience

**DOI:** 10.3389/fpubh.2025.1691496

**Published:** 2025-10-16

**Authors:** Bochuan Zhao, Haining Tu, Xinan Zhang, Yongtao Yan, Yuqi Su

**Affiliations:** ^1^School of Tourism, Physical Education and Health, Guilin University, Guilin, Guangxi, China; ^2^School of Physical Education, Shenzhen Polytechnic University, Shenzhen, China; ^3^Student Affairs Office, Guilin No. 2 Technical School, Guilin, Guangxi, China

**Keywords:** perceived social support, psychological capital, exercise self-efficacy, psychological resilience, chain mediation, firefighters

## Abstract

**Background:**

This study explored how perceived social support affects firefighters' psychological capital through the chain mediating role of exercise self-efficacy and psychological resilience.

**Methods:**

Firefighters from a Jiangsu Province fire rescue team (*N* = 524, 95.3% response rate) completed the Perceived Social Support Scale (PSSS), Self-Efficacy for Exercise Scale (SEE), Connor-Davidson Resilience Scale (CD-RISC), and Psychological Capital Questionnaire (PCQ). Hierarchical regression analysis and Bootstrap method (5,000 resamples) tested the chain mediation model.

**Results:**

The sample consisted of 99.6% males, with ages mainly concentrated between 30-45 years, and an average service length of more than 10 years. Perceived social support was significantly positively correlated with psychological capital (*r* = 0.592), exercise self-efficacy (*r* = 0.527), and psychological resilience (*r* = 0.582); exercise self-efficacy correlated with psychological resilience (*r* = 0.579; all *p* < 0.001). The chain indirect effect (perceived social support → exercise self-efficacy → psychological resilience → psychological capital) was significant [95% CI (0.09, 0.18)].

**Conclusion:**

Perceived social support enhances firefighters' psychological capital both directly and indirectly through sequential improvements in exercise self-efficacy and psychological resilience. Fire departments should create supportive team environments and integrate physical training with psychological resilience cultivation to improve firefighters' psychological capital and mental health.

## 1 Introduction

Due to their occupational nature, firefighters often face high-risk, high-pressure, and traumatic events, which pose significant challenges to their mental health (1). Research by Serrano-Ibáñez et al. (2) shows that the prevalence of psychological problems such as post-traumatic stress disorder (PTSD) and depression among firefighters is significantly higher than in the general population. A meta-analysis shows that the current global prevalence of PTSD among firefighters is ~7.3%, significantly higher than the 4.7% level among police officers (3). Another study indicates that 27.94% of firefighters experience depressive symptoms (4). Therefore, how to enhance firefighters' psychological resilience and alleviate the adverse effects of occupational stress on their mental health has become an urgent issue of concern.

Social support is widely recognized as a protective factor that improves individual mental health (5). Good social support (such as assistance from colleagues, leaders, and family members) helps individuals cope with work stress and reduce psychological distress and occupational burnout (6). Previous research has found that in firefighters and other emergency personnel, social support is closely related to mental health levels. A meta-analysis by Prati and Pietrantoni (7) found that firefighters who perceived higher levels of social support had significantly better mental health. Positive social support can not only directly buffer the negative effects of stress but also enhance individual resilience and reduce the risk of psychological pathology after trauma.

In addition to external support, firefighters' own positive psychological resources are equally important for their mental health. Among them, “psychological capital” as an important concept in positive psychology has received widespread attention. Psychological capital (PsyCap) consists of four elements: self-efficacy, hope, optimism, and resilience, representing an individual's positive psychological state (8). Bertieaux et al. point out that psychological capital is a developable psychological resource that is significantly positively correlated with work performance and wellbeing (9). For firefighters, higher psychological capital means stronger confidence and more positive attitudes when facing work challenges.. This will help reduce the negative impact of work stress and improve job satisfaction and wellbeing. According to the Conservation of Resources (COR) theory, individuals tend to acquire, maintain, and accumulate resources, and possessing one type of resource often helps in obtaining other resources (10). Social support may enhance firefighters' psychological capital by facilitating internal psychological resource development. In supportive environments, firefighters more readily develop stronger self-efficacy, hope, and psychological resilience.

Although existing research has revealed the relationships between social support, psychological resilience, and other factors with firefighters' mental health, the mechanism involved is not yet very clear (1). Exercise self-efficacy and psychological resilience may be two key mediating pathways. Exercise self-efficacy refers to an individual's confidence in persisting in physical exercise when facing various obstacle situations. Firefighters need good physical fitness to be competent in their work, and high levels of exercise self-efficacy help them maintain regular training and healthy lifestyles, thereby improving mental health. Research has found that social support can promote individual physical activity behavior by improving exercise self-efficacy (11). For firefighters, support from team comrades and organizations may increase their confidence in overcoming difficulties such as weather fatigue and sticking to training, thereby improving their physical and mental condition.

Psychological resilience refers to an individual's ability to positively adapt when facing adversity, trauma, and stress, demonstrating “springback” capacity (12). Psychological resilience has been proven to negatively predict adverse psychological outcomes such as depression and burnout (13) and is one of the core qualities for firefighters to cope with high-pressure work (14). On one hand, sufficient social support is conducive to cultivating individual psychological resilience because support systems provide resources such as emotional comfort and problem-solving, enabling individuals to “bounce back” in adversity. On the other hand, improved exercise self-efficacy, through increased physical activity and positive experiences, helps enhance psychological resilience (15). Therefore, it can be speculated that social support may first enhance firefighters' exercise self-efficacy, promote them to maintain good physical fitness and healthy habits, and thus further strengthen their psychological resilience. Psychological capital, as a higher-level positive psychological state, is likely to be comprehensively influenced by the above factors: the external resources enhanced by social support, through the layer-by-layer transmission of exercise self-efficacy and psychological resilience, ultimately manifest in the enhancement of psychological capital.

In summary, based on the Conservation of Resources theory, this study proposes a chain mediation model to explore how perceived social support affects firefighters' psychological capital and the mediating role of exercise self-efficacy and psychological resilience. We focus on the firefighter population in Jiangsu Province, collect data through questionnaire surveys, and test the following hypotheses:

H1: Perceived social support is positively correlated with psychological capital.

H2: Perceived social support is positively correlated with exercise self-efficacy.

H3: Perceived social support is positively correlated with psychological resilience.

H4: Exercise self-efficacy is positively correlated with psychological resilience.

H5: Exercise self-efficacy and psychological resilience play a chain mediating role between social support and psychological capital.

## 2 Materials and methods

### 2.1 Participants and procedure

This study used a combination of convenience sampling and random sampling to select firefighters from a fire rescue team in Jiangsu Province as participants. A single standardized questionnaire was administered to 550 firefighters. Of the 550 questionnaires distributed, 532 were returned. After data screening, 8 questionnaires were excluded due to: (1) incomplete responses with more than 10% missing data (*n* = 3), (2) short completion times (< 200 s) or obvious response patterns suggesting lack of attention (e.g., selecting the same response for all items; *n* = 5). This resulted in 524 valid questionnaires for analysis (effective response rate: 95.3%). The sample consisted of 522 males (99.6%) and two females (0.4%). Ages ranged from 18 to 50 years, mainly concentrated between 30 and 45 years; by category, 31–40 years accounted for ~60%, 26–30 years accounted for ~13%, and over 41 years accounted for ~32%. The average service length of firefighters was more than 10 years (of which over 10 years of service accounted for 58.6%, 5–10 years accounted for 10.3%, and under 5 years accounted for 31.1%). In terms of education level, high school or secondary school education was the majority, with a few having college education or above. All participants provided informed consent and voluntarily completed questionnaires. The study obtained ethical review approval from the research institution.

### 2.2 Measures

Based on preliminary research and questionnaire design, the survey questionnaire consisted of the following scales:

Perceived Social Support Scale: The adapted Chinese version of the Perceived Social Support Scale (PSSS) was used (16). This scale contains 12 items, assessing the support individuals receive from family, friends, and other significant others (such as leaders, colleagues, etc.), for example, “When I encounter difficulties, some people will appear by my side to help me.” Each item uses a seven-point Likert scale, with one representing “completely disagree” and seven representing “completely agree.” Higher scores indicate higher levels of perceived social support. In this study, the internal consistency reliability coefficient α = 0.98 for the perceived social support scale.

Self-Efficacy for Exercise Scale: The adapted Chinese version of the Self-Efficacy for Exercise Scale was used (17). This scale assesses firefighters' confidence in persisting in exercise under different obstacle situations. The scale contains 9 hypothetical situation items, such as “Continue exercising when you feel tired.” Each item uses a 0–10 point scale, with 0 representing “completely no confidence” and 10 representing “very confident.” The sum of the nine situation scores is taken as the total score, with higher total scores indicating stronger exercise self-efficacy. The Cronbach's α coefficient for this scale in this study was 0.97.

Psychological Resilience Scale: The Chinese version of the Connor-Davidson Resilience Scale (CD-RISC) compiled by Connor and Davidson was used to measure firefighters' psychological resilience levels (18). The scale has 25 items, such as “I can handle whatever happens” and “I can recover quickly after setbacks.” Participants evaluate based on their actual situation in the past month, using a five-point Likert scale (one = “never,” five = “almost always”). The average of all item scores represents the overall psychological resilience score, with higher scores indicating stronger resilience. The Cronbach's α coefficient for the psychological resilience scale in this study was 0.98.

Psychological Capital Questionnaire: The Chinese version of the Psychological Capital Questionnaire (PCQ) was used to measure firefighters' psychological capital (19). The PCQ contains 24 items, covering four dimensions: self-efficacy, hope, optimism, and psychological resilience, with six items for each dimension, such as “I believe I can find ways to solve difficulties at work” (self-efficacy dimension), “When encountering setbacks at work, I will still persevere” (resilience dimension), “I am optimistic about the future of work” (optimism dimension), etc. The scale uses a six-point Likert scale (one = “strongly disagree,” six = “strongly agree”). The average of item scores gives the overall level of psychological capital, with higher scores indicating richer positive psychological capital. In this study, the Cronbach's α coefficient for the psychological capital scale was 0.99. It is important to note that while both the CD-RISC and the resilience dimension of the PCQ measure aspects of resilience, they capture distinct constructs. The CD-RISC assesses general psychological resilience across life domains, focusing on adaptability and recovery from adversity. In contrast, the PCQ's resilience dimension specifically measures work-related resilience beliefs and recovery from occupational setbacks.

In addition, the questionnaire also collected participants' demographic information (such as gender, age, marital status, education level, and years of service). Before formal administration, the questionnaire was pretested on a small scale to clarify item expressions and ensure no ambiguity before implementing the formal survey.

### 2.3 Statistical analysis

First, SPSS 24.0 was used to organize data and conduct descriptive statistical analysis, calculating means, standard deviations, and Pearson correlation coefficients of major variables to understand the basic relationships between firefighters' perceived social support, exercise self-efficacy, psychological resilience, and psychological capital. Second, to test the proposed chain mediation effect hypothesis, hierarchical regression analysis and Bootstrap method were used for mediation effect testing. Following the procedure proposed by Hayes (Model 6), perceived social support was used as the independent variable, psychological capital as the dependent variable, and exercise self-efficacy and psychological resilience were sequentially entered as the first and second mediating variables into the model. Specific analysis steps included: (1) Regression testing the effect of perceived social support on exercise self-efficacy; (2) While controlling for social support, testing the effect of exercise self-efficacy on psychological resilience and the direct effect of social support on psychological resilience; (3) While controlling for both social support and exercise self-efficacy, testing the effect of psychological resilience on psychological capital and the direct effects of social support and exercise self-efficacy on psychological capital. The significance of partial regression coefficients and changes in *R*^2^ were used to judge the effectiveness of each regression step. Finally, the bias-corrected non-parametric percentile Bootstrap method (5,000 resamples) was used to calculate indirect effects of each mediating pathway and their 95% confidence intervals. If confidence intervals do not include 0, the indirect effect is considered significant. All statistical tests used two-tailed tests, with significance level set at α = 0.05.

## 3 Results

### 3.1 Descriptive statistics and correlation analysis

Table 1 presents the means, standard deviations, and correlation coefficients of the main variables. Overall, Jiangsu firefighters' perceived social support scores were relatively high, exercise self-efficacy and psychological capital scores were also at moderate to high levels, while psychological resilience scores were slightly above the median. For example, the average perceived social support score was 5.21 (out of 7), indicating that the sample generally felt they had adequate support; the average total exercise self-efficacy score was 58.81 (out of 90), indicating moderate to high exercise confidence when encountering some difficult situations; the average psychological resilience score was 3.60 (out of 5), and the average psychological capital score was 4.42 (out of 6), showing that firefighters' positive psychological qualities were generally at a good level.

**Table 1:** Descriptive statistics and correlations of main variables (*N* = 524).

**Variables**	**M**	**SD**	**1**	**2**	**3**
1. Perceived social support	5.21	1.47	1		
2. Exercise self-efficacy	58.81	25.50	0.527^***^	1	
3. Psychological resilience	3.60	1.00	0.582^***^	0.579^***^	1
4. Psychological capital	4.42	1.22	0.592^***^	0.589^***^	0.831^***^

^***^p < 0.001. M, mean, SD, standard deviation.

All variables showed significant positive correlations with each other (see Table 1). Among them, perceived social support was significantly positively correlated with psychological capital (*r* = 0.592, *p* < 0.001), supporting hypothesis 1; perceived social support was significantly positively correlated with exercise self-efficacy (*r* = 0.527, *p* < 0.001), supporting hypothesis 2; perceived social support was significantly positively correlated with psychological resilience (*r* = 0.582, *p* < 0.001), supporting hypothesis 3. Exercise self-efficacy was significantly positively correlated with psychological resilience (*r* = 0.579, *p* < 0.001), supporting hypothesis 4; exercise self-efficacy was also positively correlated with psychological capital (*r* = 0.589, *p* < 0.001). Notably, the correlation coefficient between psychological resilience and psychological capital was as high as 0.831 (*p* < 0.001), showing a high correlation. This is partly due to psychological capital itself containing a psychological resilience dimension, but it also confirms the important role of psychological resilience in positive psychological states.

### 3.2 Chain mediation effect testing

**Figure 1:**
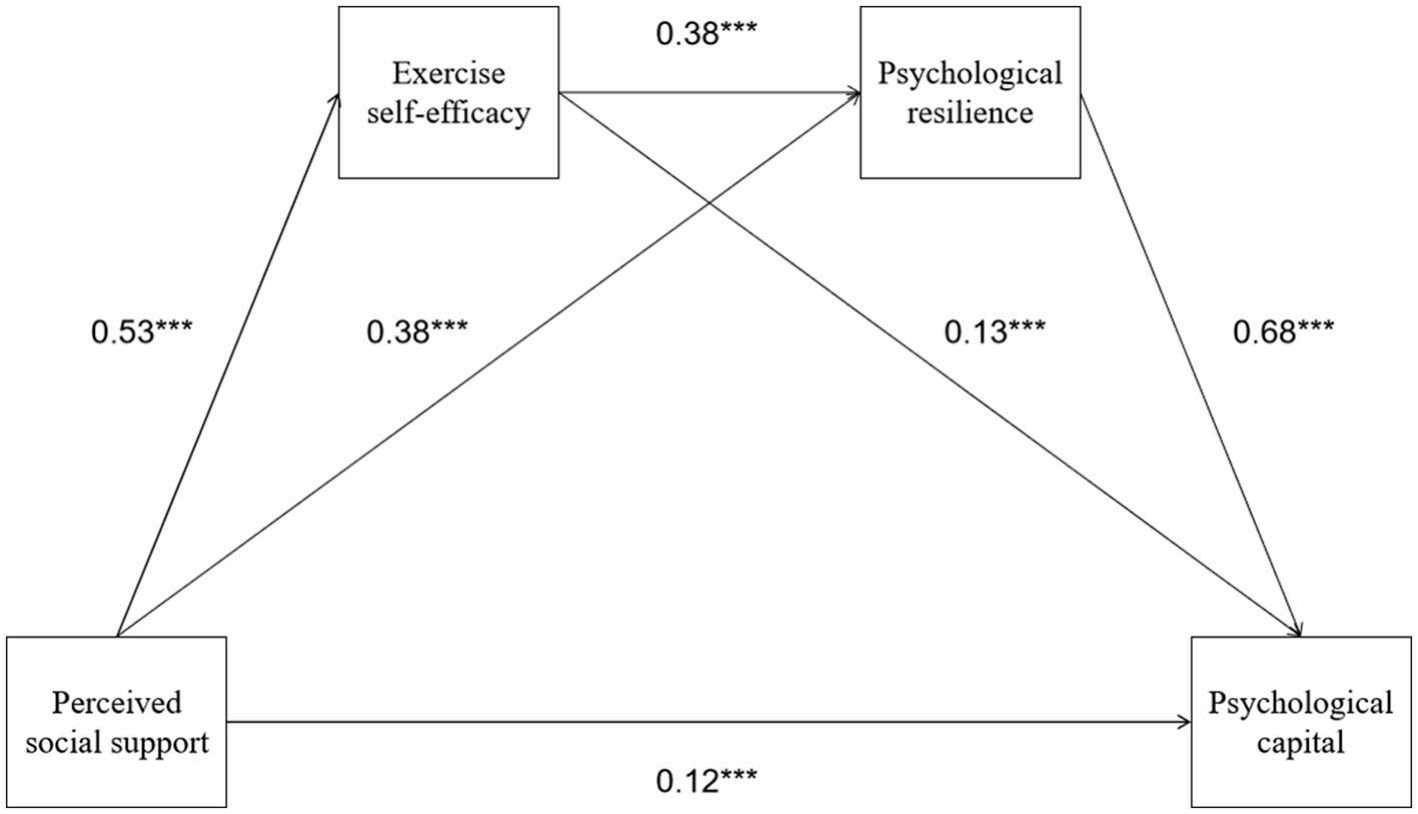
Path coefficient diagram of the chained mediation model. ***p < 0.001.

To test the chain mediation model proposed in hypothesis 5, this study conducted a series of regression analyses following the aforementioned steps. Results showed (see Table 2, Figure 1): In the first regression step, perceived social support had a significant positive predictive effect on exercise self-efficacy (β = 0.53, *p* < 0.001), indicating that firefighters who receive more support have greater confidence in overcoming difficulties and persisting in exercise. In the second step, when psychological resilience was the dependent variable, both perceived social support and exercise self-efficacy had significantly positive regression coefficients (social support: β = 0.38, *p* < 0.001; exercise self-efficacy: β = 0.38, *p* < 0.001). This shows that exercise self-efficacy positively predicts psychological resilience when controlling for social support, while social support maintains a direct positive effect on psychological resilience when considering exercise self-efficacy. This indicates that social support can directly improve firefighters' psychological resilience and can also indirectly improve psychological resilience by enhancing exercise self-efficacy, with both pathways potentially coexisting. In the third step, in the regression analysis with psychological capital as the dependent variable, when perceived social support, exercise self-efficacy, and psychological resilience were all included in the model, all three had positive and significant regression coefficients (perceived social support: β = 0.12, *p* < 0.001; exercise self-efficacy: β = 0.13, *p* < 0.001; psychological resilience: β = 0.68, *p* < 0.001). This shows that when considering chain mediation, psychological resilience has the largest independent contribution to psychological capital, but social support and exercise self-efficacy still maintain some direct effects on psychological capital, although weakened compared to single regressions. This indicates that the effect of perceived social support on psychological capital is partially transmitted through exercise self-efficacy and psychological resilience, but is not completely mediated, and direct effects still exist, representing a partial mediation model.

**Table 2:** Regression analysis among variables in the chain mediation model.

**Outcome variable**	**Predictor variable**	***R***	***R* ^2^**	***F***	**β**	***t***
Exercise self-efficacy	Perceived social support	0.53	0.28	22.51^***^	0.53	13.93^***^
Psychological resilience	Perceived social support	0.67	0.45	41.88^***^	0.38	9.67^***^
	Exercise self-efficacy				0.38	9.71^***^
Psychological capital	Perceived social support	0.85	0.72	120.21^***^	0.12	4.14^***^
	Exercise self-efficacy				0.13	4.25^***^
	Psychological resilience				0.68	21.75^***^

^***^p < 0.001. The demographic variables are controlled as covariates.

To further determine the significance of mediation effects, this study used the Bootstrap method (5,000 resamples) to calculate indirect effect values and confidence intervals for each pathway. As shown in Table 3, the indirect effect of perceived social support → exercise self-efficacy → psychological capital was 0.07 [95% CI (0.02, 0.11)], accounting for 12.07% of the total effect, indicating that the effect transmitted through the single mediator “exercise self-efficacy” was significant but small in effect size. The indirect effect of perceived social support → psychological resilience → psychological capital was 0.26 [95% CI (0.17, 0.36)], representing the largest contribution at 44.83% of the total effect, indicating that social support largely promotes psychological capital by enhancing firefighters' psychological resilience and highlighting psychological resilience as the primary mediating mechanism. Most importantly, the chain indirect effect of perceived social support → exercise self-efficacy → psychological resilience → psychological capital had an estimated value of 0.14, with a 95% confidence interval of [0.09, 0.18], not including 0, accounting for 24.13% of the total effect. This indicates that this sequential mediation effect was significant, and firefighters' perceived social support can lead to enhanced psychological capital levels by sequentially improving their exercise self-efficacy and psychological resilience. This finding supports hypothesis 5, validating the chain mediation model of exercise self-efficacy and psychological resilience.

**Table 3:** Analysis of the mediating effect of exercise self-efficacy and psychological resilience.

**Pathway**	**Effect size**	**Standard error**	**Boot CI LL**	**Boot CI UL**	**Relative mediation effect %**
Direct effect	0.11	0.03	0.06	0.16	18.97%
Indirect effect 1	0.07	0.02	0.02	0.11	12.07%
Indirect effect 2	0.26	0.05	0.17	0.36	44.83%
Indirect effect 3	0.14	0.02	0.09	0.18	24.13%
Total mediation effect	0.58	0.04	0.51	0.65	100%

Indirect effect 1: Perceived social support → Exercise self-efficacy → Psychological capital. Indirect effect 2: Perceived social support → Psychological resilience → Psychological capital. Indirect effect 3: Perceived social support → Exercise self-efficacy → Psychological resilience → Psychological capital. The demographic variables are controlled as covariates.

In contrast, the direct effect of social support on psychological capital accounts for only 18.97% of the total effect. Together, the three indirect effects account for 81.03% of the total effect, confirming that the relationship between social support and psychological capital is primarily mediated rather than direct. These results indicate that in the firefighter population, social support affects psychological capital levels through complex mediation mechanisms, including both simple single mediation effects and sequential chain mediation effects. Although social support has a direct impact on psychological capital, its proportion is relatively small compared to the total impact, indicating that exercise self-efficacy and psychological resilience play an important partial mediating role in this process.

## 4 Discussion

This study verified the positive impact of perceived social support on psychological capital and its mechanism through a survey of Chinese firefighters. The main findings include: First, the level of social support perceived by firefighters is significantly positively correlated with their psychological capital, confirming the importance of social support as a protective resource for individuals' positive psychological states. Second, exercise self-efficacy and psychological resilience play significant mediating roles between social support and psychological capital, and both constitute a chain mediation: social support can not only directly enhance firefighters' psychological capital but also indirectly promote the growth of psychological capital by first enhancing exercise self-efficacy and then improving psychological resilience. The following will further explain these findings and compare them with existing literature.

First, the significant positive correlation between perceived social support and firefighters' psychological capital indicates that firefighters with rich social support often have higher levels of psychological capital elements such as self-efficacy, optimism, hope, and resilience. This aligns with Conservation of Resources theory and is consistent with research on general occupational populations, where external support helps individuals accumulate positive psychological resources Research shows that over time, social support at work has positive effects on psychological capital, with social support achieving self-efficacy through positive experiences of success and mastery (20). Previous research has shown that in healthcare worker populations, individuals with higher levels of perceived social support often have higher levels of psychological capital, buffering the negative effects of work stress (21). According to Conservation of Resources theory, social support is a potential resource (22). Through perceived social support, individuals can transform external social resources into internal psychological resources, thereby improving their psychological capital levels. For high-pressure, high-risk occupational groups like firefighters, social support is particularly important. Support from colleagues, leaders, and family provides firefighters with emotional security and a sense of value. This supportive environment encourages them to face work challenges with a more positive attitude, enhancing psychological capital components such as self-efficacy and optimistic expectations (23). Additionally, strong social support networks can reduce firefighters' feelings of helplessness and loneliness after traumatic events, thereby maintaining high levels of hope and psychological resilience. Therefore, this study confirms the close connection between social support and positive psychological qualities in the firefighter population, which is also consistent with previous research findings.

Second, one innovation of this study is the introduction of “exercise self-efficacy” as a specific mediating variable, revealing how social support indirectly affects psychological capital by influencing firefighters' confidence in healthy behaviors. Results show that firefighters with high levels of perceived social support often have stronger exercise self-efficacy, and the improvement of exercise self-efficacy helps enhance firefighters' psychological resilience. This pathway is supported by research results and is consistent with research in related fields. In general populations, research has found that social support can enhance individuals' exercise self-efficacy, such as peer and school support increasing college students' exercise confidence by improving their exercise confidence, leading to increased physical activity behavior and promoting long-term persistence in physical activity (11). For firefighters, mutual support and encouragement within teams (such as training together and role modeling) may also improve their belief in overcoming various difficulties and persisting in training (24, 25). When firefighters believe they can persist in physical training despite fatigue, stress, or adverse weather conditions, they are more likely to maintain good physical fitness and healthy habits. Such regular exercise not only strengthens physical fitness but also brings psychological benefits—numerous studies show that moderate physical exercise can reduce anxiety and depression, improve cognitive function, and enhance stress resistance (26, 27). Therefore, improved exercise self-efficacy reflects enhanced control over health behaviors, embodying the self-efficacy and hope elements of psychological capital. More importantly, we found that exercise self-efficacy further promotes firefighters' psychological resilience. The positive experience of persisting in exercise may give firefighters more confidence to face physical and psychological challenges at work, thus showing stronger resilience in adversity. This finding extends previous research that only focused on the direct effect of social support on psychological resilience, indicating that enhancing resilience through exercise pathways is an important mediation mechanism not to be ignored (28, 29). This has instructive significance in practical interventions: while emphasizing emotional support is important, combining this with enhancing firefighters' own exercise confidence and behavior may more effectively cultivate their psychological resilience and overall psychological capital.

Third, psychological resilience plays a key mediating role in the process of social support affecting psychological capital. Correlation analysis shows that psychological resilience and psychological capital are highly correlated, which is largely due to the conceptual connection between them: psychological capital includes resilience but is not completely equivalent to resilience (30). Regression analysis further shows that even when considering social support and exercise self-efficacy simultaneously, psychological resilience still has the strongest predictive effect on psychological capital. This means that firefighters' psychological resilience level is crucial for their overall psychological capital level. A considerable portion of social support's impact on psychological capital is achieved by enhancing firefighters' psychological resilience. This study supports this view because the indirect effect of “social support → psychological resilience → psychological capital” is significant and has a large effect size. This result is consistent with existing literature. Zhang et al.'s (31) research emphasizes that resilience plays a mediating or buffering role between stress and psychological adaptation, with highly resilient people maintaining more positive psychological states in adversity. For firefighters, psychological resilience not only means rapid recovery at disaster scenes and in high-pressure environments but also represents a positive coping orientation and belief. Therefore, viewing psychological resilience as a bridge for social support to help firefighters accumulate psychological capital is reasonable. Firefighters with sufficient support are less likely to give up during setbacks, thus demonstrating greater psychological capital. The last link in this chain—the effect of psychological resilience on psychological capital—also confirms Luthans et al.'s (32) view on psychological capital theory: psychological capital as a whole composed of multiple components can better predict individual performance than each component individually. In this study, we also see that when firefighters have high levels of psychological resilience, their self-efficacy, optimism, and other positive psychological qualities also tend to rise, with overall psychological capital being abundant.

This study has certain theoretical contributions. First, we constructed and validated a chain mediation model based on Conservation of Resources theory in the firefighter population, deepening our understanding of how social support transforms into individual positive psychological resources. Second, this study enriches the application research of psychological capital theory by combining the special occupational background of firefighters. Simultaneously, our research confirms the “chain reaction” of resource acquisition in stressful situations: having social support as a resource helps individuals gain subsequent resources such as self-efficacy and resilience, with resource gains showing cumulative effects. These findings provide new empirical evidence for the expansion of positive psychology in the emergency rescue field.

The findings of this study also have important practical implications. First, the results emphasize the importance of creating a good social support environment for firefighters' mental health and positive psychological qualities. Fire department managers should value team atmosphere building, advocate mutual assistance and cooperation among comrades, and establish formal or informal peer support networks. Second, attention should be paid to intervention strategies combining “physical fitness + psychology,” utilizing the role of exercise self-efficacy in improving psychological resilience. Additionally, professional fitness coaches or exercise psychology experts can be invited to provide training on exercise psychological skills to team members, helping them learn techniques for setting exercise goals, self-motivation, and overcoming inertia. Third, direct cultivation of psychological resilience is also indispensable. Firefighters' resilience depends not only on daily exercise and supportive environments but can also be strengthened through specialized psychological training and counseling. Through the above multi-level interventions, firefighters' psychological resilience levels are expected to steadily improve and ultimately transform into higher psychological capital and better mental health.

Of course, this study also has some limitations. First, the study uses a cross-sectional survey design, which cannot strictly infer causal relationships. Future research could further verify the causal chain of variables through longitudinal tracking or experimental intervention methods. Second, all data come from firefighters' self-reports, which inevitably have the possibility of common method bias. Future research could consider obtaining information about social support and psychological capital from multiple sources such as colleague or supervisor evaluations, or introduce objective indicators for corroboration. Third, sample characteristics limit generalizability. The sample consists almost entirely of male participants (99.6%), comes exclusively from Jiangsu Province firefighters, and represents a specific cultural and occupational context. Future studies should include more diverse samples across gender, regions, and organizational settings to test the model's robustness. Fourth, the conceptual overlap between the elastic dimensions of CD-RISC and PCQ may exaggerate their correlation coefficient (*r* = 0.831). While the CD-RISC assesses general life resilience and the PCQ focuses on work-specific psychological resources, this structural overlap in measurement content should be acknowledged. Future studies should consider using measurement tools with greater conceptual independence. Finally, this study focused on exercise self-efficacy and psychological resilience as mediating mechanisms, but other important factors warrant exploration, including leadership style, individual coping strategies, organizational climate, and team cohesion. Future research should examine these additional pathways to provide a more comprehensive understanding of how social support enhances psychological capital in firefighters.

## 5 Conclusion

In summary, this study focused on firefighters as a high-risk occupational population, revealing the chain mediation mechanism of perceived social support affecting their psychological capital through exercise self-efficacy and psychological resilience. Research results emphasize that sufficient social support can not only directly enhance firefighters' psychological capital but also indirectly produce positive effects by enhancing their confidence in persisting in exercise and further improving psychological resilience. This finding provides new insights for improving firefighters' mental health: while focusing on team support and care, encouraging and helping firefighters enhance their exercise self-efficacy and psychological resilience can effectively strengthen their psychological capital, thereby improving overall mental health levels and occupational competence. The theoretical and practical significance of this study lies in combining social support, health behavior cognition, and positive psychological qualities, enriching our understanding of the influencing factors and mechanisms of firefighters' mental health, and providing empirical evidence for developing psychological intervention and training programs for firefighters.

## Data Availability

The raw data supporting the conclusions of this article will be made available by the authors, without undue reservation.

## References

[1] Lowery A Cassidy T. Health and well-being of first responders: the role of psychological capital, self-compassion, social support, relationship satisfaction, and physical activity. J Workplace Behav Health. (2022) 37:87–105. 10.1080/15555240.2021.1990776

[2] Serrano-Ibáñez ER Corrás T Del Prado M Diz J Varela C. Psychological variables associated with post-traumatic stress disorder in firefighters: a systematic review. Trauma Violence Abuse. (2023) 24:2049–66. 10.1177/15248380221082944 35521996 PMC10486174

[3] Berger W Coutinho ES Figueira I Marques-Portella C Luz MP Neylan TC. Rescuers at risk: a systematic review and meta-regression analysis of the worldwide current prevalence and correlates of PTSD in rescue workers. Soc Psychiatry Psychiatr Epidemiol. (2012) 47:1001–11. 10.1007/s00127-011-0408-2 21681455 PMC3974968

[4] Sun X Li X Huang J An Y. Prevalence and predictors of PTSD, depression and posttraumatic growth among Chinese firefighters. Arch Psychiatr Nurs. (2020) 34:14–8. 10.1016/j.apnu.2019.12.007 32035583

[5] Acoba EF. Social support and mental health: the mediating role of perceived stress. Front Psychol. (2024) 15:1330720. 10.3389/fpsyg.2024.1330720 38449744 PMC10915202

[6] Liu Y Aungsuroch Y. Work stress, perceived social support, self-efficacy and burnout among Chinese registered nurses. J Nurs Manag. (2019) 27:1445–53. 10.1111/jonm.12828 31306524

[7] Prati G Pietrantoni L. The relation of perceived and received social support to mental health among first responders: a meta-analytic review. J Community Psychol. (2010) 38:403–17. 10.1002/jcop.20371

[8] Yuan Z Zhang X Wang F Jin M Teng M He H. Levels of psychological capital among nurses: a systematic review and meta-analysis. Int Nurs Rev. (2023) 70:89–96. 10.1111/inr.12803 36205604

[9] Bertieaux D Hesbois M Goyette N Duroisin N. Psychological capital and well-being: an opportunity for teachers' well-being? Scoping review of the scientific literature in psychology and educational sciences. Acta Psychologica. (2024) 248:104370. 10.1016/j.actpsy.2024.104370 38943874

[10] Egozi Farkash H Lahad M Hobfoll SE Leykin D Aharonson-Daniel L. Conservation of resources, psychological distress, and resilience during the COVID-19 pandemic. Int J Public Health. (2022) 67:1604567. 10.3389/ijph.2022.1604567 36119444 PMC9472268

[11] Zhang Y Hasibagen Zhang C. The influence of social support on the physical exercise behavior of college students: the mediating role of self-efficacy. Front Psychol. (2022) 13:1037518. 10.3389/fpsyg.2022.1037518 36532973 PMC9756807

[12] Labrague LJ. Psychological resilience, coping behaviours and social support among health care workers during the COVID-19 pandemic: a systematic review of quantitative studies. J Nurs Manag. (2021) 29:1893–905. 10.1111/jonm.13336 33843087 PMC8250179

[13] González-Flores CJ García-García G Lerma A Pérez-Grovas H Meda-Lara RM Guzmán-Saldaña RME. Resilience: a protective factor from depression and anxiety in Mexican dialysis patients. Int J Environ Res Public Health. (2021) 18:11957. 10.3390/ijerph182211957 34831713 PMC8620979

[14] Han YR Yun JA Jeong KS Ahn YS Choi KS. Posttraumatic stress disorder symptoms and neurocognitive functioning in fire fighters: the mediating role of sleep problems and resilience. Compr Psychiatry. (2021) 109:152250. 10.1016/j.comppsych.2021.152250 34116367

[15] Neumann RJ Ahrens KF Kollmann B Goldbach N Chmitorz A Weichert D. The impact of physical fitness on resilience to modern life stress and the mediating role of general self-efficacy. Eur Arch Psychiatry Clin Neurosci. (2022) 272:679–92. 10.1007/s00406-021-01338-9 34622343 PMC9095527

[16] Guo K Zhang X Bai S Minhat HS Nazan AINM Feng J. Assessing social support impact on depression, anxiety, and stress among undergraduate students in Shaanxi province during the COVID-19 pandemic of China. PLoS ONE. (2021) 16:e0253891. 10.1371/journal.pone.0253891 34297731 PMC8301624

[17] Wong EML Leung DYP Sit JWH Chan AWK Chair SY. Prospective validation of the Chinese version of the self-efficacy for exercise scale among middle-aged patients with coronary heart disease. Rehabil Nurs. (2020) 45:74–9. 10.1097/RNJ.0000000000000156 32118864

[18] Sharif-Nia H Sánchez-Teruel D Sivarajan Froelicher E Hejazi S Hosseini L Khoshnavay Fomani F. Connor-Davidson resilience scale: a systematic review psychometrics properties using the COSMIN. Ann Med Surg. (2024) 86:2976–91. 10.1097/MS9.0000000000001968 38694299 PMC11060289

[19] Dirzyte A Perminas A Biliuniene E. Psychometric properties of satisfaction with life scale (SWLS) and psychological capital questionnaire (PCQ-24) in the Lithuanian population. Int J Environ Res Public Health. (2021) 18:2608. 10.3390/ijerph18052608 33807777 PMC7967519

[20] Kerksieck P Bauer GF Brauchli R. Personal and social resources at work: reciprocal relations between crafting for social job resources, social support at work and psychological capital. Front Psychol. (2019) 10:2632. 10.3389/fpsyg.2019.02632 31824390 PMC6883902

[21] He Y Wang D Wang L Liao R. Latent profiles of psychological capital in clinical nursing teachers and their association with the practice environment of nursing and perceived social support. Front Psychol. (2025) 16:1527252. 10.3389/fpsyg.2025.1527252 40309212 PMC12041037

[22] Schiff M Pat-Horenczyk R Benbenishty R. Using conservation of resources theory to explain university students' anxiety, depression, and learning experience during COVID-19. J Am Coll Health. (2025) 73:1815–24. 10.1080/07448481.2024.2447854 39928024

[23] Safi F Areshtanab HN Ghafourifard M Ebrahimi H. The association between self-efficacy, perceived social support, and family resilience in patients undergoing hemodialysis: a cross-sectional study. BMC Nephrol. (2024) 25:207. 10.1186/s12882-024-03629-4 38918709 PMC11202372

[24] Chen J Li W An Y Zhang Y Du J Xu W. Perceived social support mediates the relationships of dispositional mindfulness to job burnout and posttraumatic stress disorder among Chinese firefighters. Psychol Trauma. (2022) 14:1117–23. 10.1037/tra0000534 31750689

[25] Wang L Chen F Zhang Y Ye M. Association between social support, and depressive symptoms among firefighters: the mediating role of negative coping. Saf Health Work. (2023) 14:431–7. 10.1016/j.shaw.2023.10.002 38187206 PMC10770279

[26] Fu HY Wang J Hu JX. Influence of physical education on anxiety, depression, and self-esteem among college students. World J Psychiatry. (2023) 13:1121–32. 10.5498/wjp.v13.i12.1121 38186731 PMC10768485

[27] Endeshaw Y Goldstein F. Association between physical exercise and cognitive function among community-dwelling older adults. J Appl Gerontol. (2021) 40:300–9. 10.1177/0733464820952242 32844709

[28] Deng D Sun Q Li H. The influences of physical exercise on student burnout: based on the mediating role of psychological resilience. BMC Psychol. (2025) 13:114. 10.1186/s40359-025-02394-9 39934909 PMC11817967

[29] Beitia P Stamatis A Amasay T Papadakis Z. Predicting firefighters' physical ability test scores from anaerobic fitness parameters & mental toughness levels. Int J Environ Res Public Health. (2022) 19:15253. 10.3390/ijerph192215253 36429971 PMC9691205

[30] Zhang R Shan M Yin Y Wu Y Gao P Xin Y. Psychological capital appreciation as a mediator between resilience and burnout among ICU nurses. Front Public Health. (2025) 13:1551725. 10.3389/fpubh.2025.1551725 40308908 PMC12040887

[31] Zhang Y Zhang X Zhang L Guo C. Executive function and resilience as mediators of adolescents' perceived stressful life events and school adjustment. Front Psychol. (2019) 10:446. 10.3389/fpsyg.2019.00446 30873099 PMC6403185

[32] Luthans F Youssef-Morgan CM. Psychological capital: an evidence-based positive approach. Annu Rev Organ Psychol Organ Behav. (2017) 4:339–66. 10.1146/annurev-orgpsych-032516-113324

